# Unexpected viscoelastic deformation of tight sandstone: Insights and predictions from the fractional Maxwell model

**DOI:** 10.1038/s41598-017-11618-x

**Published:** 2017-09-12

**Authors:** Xiang Ding, Guangqing Zhang, Bo Zhao, Yan Wang

**Affiliations:** 1 College of Petroleum Engineering, China University of Petroleum, 102249 Beijing, China; 2 State Key Laboratory of Petroleum Resources and Engineering, 102249 Beijing, China

## Abstract

Tight gas is one important unconventional hydrocarbon resource that is stored in tight sandstone, whose mechanical property greatly influences the tight gas production process and is commonly believed to be simply elastic when designing the stimulation plan. However, the experimental evidence provided in this work surprisingly shows that tight sandstone can deform in a viscoelastic way. Such an unexpected observation poses a challenge in accurately modelling the deformation process. We solve this problem by adopting the fractional Maxwell model to successfully derive the constitutive equation of tight sandstone, based on which not only all the experimental data can be interpreted quantitatively, but also reasonable and consistent predictions as to tight sandstone’s long-term deformation behaviour can be made. We then investigate the typicality of our results in China’s Changqing oilfield, which is one major centre of tight gas production and where the rock samples for experiments are obtained. It is estimated that a non-negligible portion of 18% tight sandstone samples in this area will probably display viscoelasticity. Finally, our work implies that the mechanical properties of other materials may also need further scrutiny to possibly uncover any unexpected behaviour, overlooking which may result in misleading predictions.

## Introduction

Unconventional hydrocarbon resources are nowadays playing an important role in the global energy industry^1^. As one major constituent of them, the so-called tight gas is successfully exploited in many regions of the world by hydraulic fracturing, a technology that creates artificial fractures in low permeability reservoirs by injecting high pressure fluids into the reservoir rock^2^. These fractures serve as pathways for gas flow, and the maintenance of such fractures under the *in situ* stress condition is of paramount significance to the long-term recovery of tight gas. That is, most of the fractures should not close during the whole production process that typically lasts for some tens of years. The closure of fractures in tight gas reservoirs is determined by the mechanical response of tight sandstone^3^. A natural question thus arises that, once created, to what extent will the fractures close due to the deformation of tight sandstone, especially in a relatively long period of time? It is a common way to simply model the sandstone as an elastic body when designing the plan of hydraulic fracturing in which the elasticity parameters (such as the Young’s modulus *E*) are obtained using conventional tri-axial experiments and then fed into the simulator. A tacit assumption here is that these parameters do not vary appreciably with time, i.e., the tight sandstone is purely elastic. This assumption is plausible partly due to the naïve perception that the tight sandstone is hard and brittle, and should behave in a simple way. Assuming this may not cause any big problem in designing the stimulation plan because the hydraulic fracturing process only takes a very small amount of time as compared with the whole production process. However, are these parameters really time independent? Or, is the deformation behaviour of tight sandstone really entirely elastic? Despite many operations of hydraulic fracturing in tight sandstone reservoirs, to date and to the best of our knowledge, there seem to be few studies addressing this issue, and there is no conclusive or convincingly supportive evidence that tight sandstone can be modelled as an elastic body in a time span of several years. It is one of our central tasks here to investigate whether such an assumption is generally valid.

To this end, we perform a series of creep experiments for tight sandstone samples, which are obtained from the Changqing oilfield, one of the major tight gas development centers in China. Our result surprisingly shows that an unexpected viscoelastic deformation can be observed for some of our rock samples, indicating the failure of the elastic assumption. Moreover, the observed deformation behaviour is even beyond the prediction of several classical viscoelastic models such as the Maxwell model, which applies to “simple” viscoelastic materials with only a single relaxation mode. In order to quantitatively explain the experimental data, we adopt the fractional Maxwell model^4–8^, which however has been successful in describing the rheological dynamics for such soft matters as polymers^9–11^, foods^12, 13^ and fluids^14^, seems not a candidate for tight sandstone modelling by far within the geomechanical science and petroleum engineering communities. One striking difference between “soft” and “hard” materials is that the latter deform in a seemingly quasi-memoryless way, i.e., their deformation is almost simultaneously finished due to a fast (compared with the loading rate) relaxation towards a quasi-equilibrium steady state. While the former deform at a finite rate, and they slowly but persistently respond to previous driving as a result of the local microstructure reorganization process that spans several spatial and temporal scales to reach equilibrium. Rocks other than shale and salt rock are commonly considered as hard. Nevertheless, based on this fractional Maxwell model typically for “soft” materials, we find an excellent correspondence between theory and experimental results of the “hard” tight sandstone. This counter-intuitive fact implies that the relaxation dynamics of tight sandstone can be much more complex than previously thought. In particular, the strong memory effect should be taken into account to arrive at a satisfactory modelling of the mechanical response behaviour of tight sandstone. Although the exact underlying physical mechanism is still elusive to date (due to the notoriously complex nature of tight sandstone as a kind of natural composite materials usually with the presence of inhomogeneity and anisotropy), we believe the phenomenological fractional Maxwell model serves well as a way to understand the mechanical response of tight sandstone. We then use this model to predict the strain rate and the stress relaxation of rock samples in the long run, which are of great importance in assessing to what extent the fracture closure will be, and it turns out that such predictions are fully consistent with previous results. Therefore, reasonable conclusions can be made as to the durability of the hydraulic fracture system^15, 16^ as pathways for gas flow.

By presenting experimental data that demonstrate tight sandstone’s unexpected viscoelasticity and the corresponding fractional Maxwell model that quantitatively describes such a property, our work sheds new light on the understanding of tight sandstone’s mechanical response behaviour. Our findings also provide useful insights into the fracture closure problem, and hopefully will help with the development plan, as well as the profitability assessment, of a tight gas reservoir. Moreover, in light of this work, we call for a careful scrutiny of the mechanical response behaviour of other materials. For example, more testing experiments performed to other kinds of reservoir rock are suggested to possibly uncover some counter-intuitive property just like tight sandstone’s viscoelasticity, which can be pivotal for an accurate long-term prediction of oil recovery, but has long been, and will otherwise continuously be overlooked by both academia and industry.

## Results

### Experimental results

**Figure 1:**
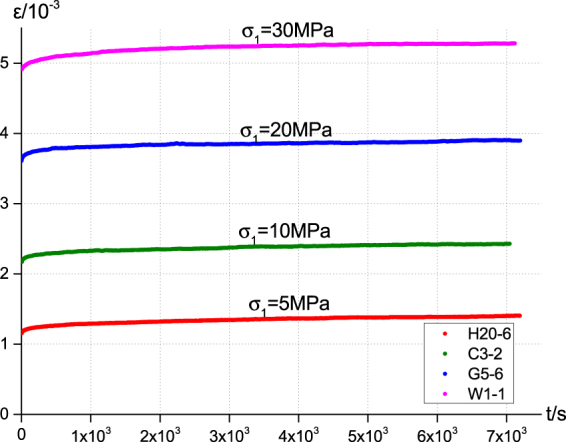
Experimentally obtained axial strains (ε) of four tight sandstone samples (denoted as H20-6, C3-2, G5-6, W1-1, respectively) under corresponding axial stresses (σ) are plotted versus the experiment time. In each case, ε is found to be time dependent, indicating the creep nature of the sample’s deformation behaviour.

We perform standard uniaxial compression creep experiments for four tight sandstone samples obtained from the Changqing oilfield. The corresponding axial stress for each sample ranges from 5 MPa to 30 MPa. (Experimental details are given in Methods). As shown in Fig. 1, in each case, the axial strain of the sample ε increases with time *t* as the axial stress σ is fixed. Since purely elastic deformation means a fixed strain under a given stress condition, the experimental observations thus reject the assumption that such tight sandstone samples can be effectively modelled as an elastic body. Rather, the time dependent strains clearly signal the creep nature of these samples’ deformation behaviour that is to be quantitatively described in the following.

### Fitting experiment data to the fractional Maxwell model

Besides its role in determining the effectiveness of the hydraulic fracture system as pathways for tight gas flow, creep is a central issue in many other geoscience and petroleum engineering related problems. For example, it may cause reservoir compaction, resulting in damage to wellbores, the overlying caprock and natural fault/seal systems^17^. Creep of rock on a geological scale is still poorly understood presently, and there are unresolved debates over the creep rate of rock under certain geological conditions^18^. In many cases, it is even practically prohibitive to accurately predict the creep rate due to scant data. Nonetheless, a reasonable model that is both capable of interpreting experimental results and consistent with previous independent findings seems to provide useful information as to the rock deformation process that is otherwise intractable. In this work, we develop a reliable quantitative description of the creep behaviour of tight sandstone by fitting our experimental data to the fractional Maxwell model.

**Figure 2:**
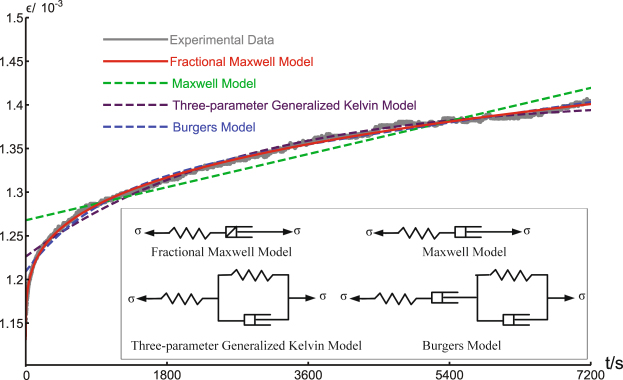
Fitting experimental data of the H20-6 sample to four rheological models. The Maxwell model (with two parameters) predicts a constant creep rate, however, the apparent nonlinear evolution of ε indicates the failure of the Maxwell model. Three-parameter generalized Kelvin (with three parameters) and Burgers (with four parameters) models perform increasingly better since they are capable of describing more complex dynamics with increasingly more parameters. However, the prediction based on the fractional Maxwell model agrees best with the experimental data in the full range of time, and there are only a moderate number of parameters (three) in this model, implying the model’s success in describing the underlying physics of the creep behaviour of tight sandstone.

There have been some widely used models for creep, such as the Maxwell model^19^, the three-parameter generalized Kelvin model^20^, and the Burgers model^21^, each of which has been successful in dealing with some time dependent physical problems^22–31^. The Maxwell model can be considered as a minimal rheological model suitable for physical processes that are governed by just one underlying relaxation mode. That is, it describes the deformation behaviour with only a single characteristic time scale *τ*_*c*_, and such a deformation process consequently manifests itself as a constant strain rate and an exponential type of stress relaxation. Graphically, the Maxwell model is composed of a Hooke spring (serving as the driving elastic force) and a Newton dashpot (representing the damping), as illustrated in the inset of Fig. 2. The Maxwell model can be modified to take more relaxation modes and/or more complex physical interactions into consideration; graphically this is represented by adding more Hooke springs and/or Newton dashpots to the traditional Maxwell model. The three-parameter generalized Kelvin model consists of two Hooke springs and one Newton dashpot, and the Burgers model is constructed by two Hooke springs and two Newton dashpots (see the inset of Fig. 2). With more elements, the generalized models may fit the data accurately, but at the expense of becoming more complicated formally. There are more model parameters need to be estimated numerically. If the underlying creep dynamics contains multiple modes, then from a statistical inference point of view, the task of parameter estimation itself can become awkwardly prohibitive, let alone the selection of a proper model.

Alternatively, the fractional Maxwell model seems to be a promising candidate for describing complex creep dynamics. It is qualitatively different from the Maxwell model, as well as any model that is graphically constructed by a finite combination of Hooke springs and Newton dashpots. The fractional Maxwell model can be simply constructed by only replacing the Newton dashpot in the Maxwell model by a Scott Blair dashpot^32^ (sometimes also referred to as Abel dashpot^33^ or spring-pot^34, 35^), which is a fractional order dashpot with the physical meaning of history dependent damping. In theory, the fractional Maxwell model is suitable for describing the behaviour of a system with an infinite number of relaxation modes, and by adopting this model for data fitting, only three model parameters need to be estimated. Note that the Maxwell model contains two parameters, thus the extra one parameter in the fractional Maxwell model plays a paramount role in accounting for the history dependent damping effect. In fact, the fractional Maxwell model is reminiscent of the fractional advection-diffusion equations, which are found to be successful in quantitatively interpreting experimental and numerical results of transport in porous media, also by simply introducing a fractional order of time derivative to the classical advection-diffusion equation. In some sense, all such fractional counterparts with one more parameter (the time fractional order) serve as a minimal model for the corresponding history dependent dynamics. Meanwhile, it is also practically feasible to handle these fractional approaches with just one more parameter than the classical models.

It is worth noting that there are other types of fractional models^36, 37^ which might also be used to interpret the experimental data, such as the fractional Kelvin-Voigt model, which has the same level of simplicity as the fractional Maxwell model does, and the more sophisticated Giusti-Colombaro model^38^, from which either the fractional Maxwell or the fractional Kelvin-Voigt model can be obtained as a much simplified special case. In particular, the fractional Kelvin-Voigt model is sometimes believed to be suitable for the viscoelastic solid, whereas the fractional Maxwell model for the viscoelastic liquid^39^. However, as a kind of natural composite, tight sandstone may be too complex to be fully represented by one single model. In fact, its deformation dynamics as stated above is still poorly understood to date. Moreover, since our major concern in this work is to effectively capture the main features of the observed unexpected time-dependent deformation process of tight sandstone, rather than to understand this behaviour from a fundamental level of physics, we do not intend to select a “best” fractional model. Actually, this task may not be easily achieved without more available data. (We have indeed compared the result from the fractional Maxwell model and that from the fractional Kelvin-Voigt model based on our experimental data, but no decisive conclusion can be made as to which fractional model is intrinsically more proper.) From a somewhat practical point of view, the fractional Maxwell model as a feasible approach serves well to our purpose, which, by taking into account the memory effect, better describes the deformation process qualitatively and quantitatively than classical models.

Concretely, we will show in the following that the fractional Maxwell model is superior to all other three classical models with increasing model parameters. (As listed in Table 1, the Maxwell model has two parameters, the three-parameter generalized Kelvin model has three, and the Burgers model has four). The superiority of the fractional Maxwell model is even visually evident in Fig. 2.

**Table 1:** Fitting of creep compliance *J*(*t*) with different rheological models.

Model	*J*(*t*)	Parameters	*R* ^2^
Fractional Maxwell model	$\frac{1}{{E}_{{\rm{H}}}}+\frac{1}{{\eta }_{{\rm{A}}}^{\alpha }}\frac{{t}^{\alpha }}{{\rm{\Gamma }}(1+\alpha )}$	*E*_H_ = 4.426 GPa	0.99
		$${\eta }_{{\rm{A}}}^{\alpha }=219.562\,{\rm{GPa}}\cdot {s}^{\alpha }$$	
		*α* = 0.268	
Maxwell model	$\frac{1}{{E}_{{\rm{H}}}}+\frac{t}{{\eta }_{{\rm{N}}}}$	${E}_{{\rm{H}}}=3.944\,{\rm{GPa}}$	0.99
		$${\eta }_{{\rm{N}}}=2.375\times {10}^{5}\,{\rm{GPa}}\cdot {\rm{s}}$$	
Three-parameter generalized Kelvin model	$\frac{1}{{E}_{{\rm{H}}}}+\frac{1}{{E}_{{\rm{K}}}}[1-\exp (-\frac{{E}_{{\rm{K}}}}{{\eta }_{{\rm{K}}}}t)]$	${E}_{{\rm{H}}}=4.078\,{\rm{GPa}}$	0.99
		$${E}_{{\rm{K}}}=27.741\,{\rm{GPa}}$$	
		$${\eta }_{{\rm{K}}}=7.481\times {10}^{4}\,{\rm{GPa}}\cdot {\rm{s}}$$	
Burgers model	$\frac{1}{{E}_{{\rm{H}}}}+\frac{t}{{\eta }_{{\rm{N}}}}+\frac{1}{{E}_{{\rm{K}}}}[1-\exp (-\frac{{E}_{{\rm{K}}}}{{\eta }_{{\rm{K}}}}t)]$	${E}_{{\rm{H}}}=4.135\,{\rm{GPa}}$	0.99
		$${E}_{{\rm{K}}}=49.254\,{\rm{GPa}}$$	
		$${\eta }_{{\rm{N}}}=3.896\times {10}^{5}\,{\rm{GPa}}\cdot {\rm{s}}$$	
		$${\eta }_{{\rm{K}}}=4.667\times {10}^{4}\,{\rm{GPa}}\cdot {\rm{s}}$$	

We will omit the well-known constitutive equations for the Maxwell, three-parameter generalized Kelvin, and Burgers models, and will only briefly introduce the relevant basics of the fractional Maxwell model here. (Details are given in Methods). The constitutive equation for the fractional Maxwell model^7, 40, 41^ is

1 $$\sigma ={\eta }^{\alpha }\frac{{{\rm{d}}}^{\alpha }}{{\rm{d}}{t}^{\alpha }}(\varepsilon -\frac{\sigma }{E})$$

where *α* ∈ (0, 1) is the fractional order of the Scott Blair dashpot, *E* is the elastic modulus of the Hooke spring, *η* ^*α*^ is the generalized viscosity of the Scott Blair dashpot, and $\frac{{{\rm{d}}}^{\alpha }}{{\rm{d}}{t}^{\alpha }}$ indicates fractional differentiation. Fractional calculus can be defined in different ways^7, 42–45^, such as the Riemann-Liouville and Caputo calculus. However, in this work we are dealing with initial conditions that will make no difference between these two commonly adopted methods of differentiation^35, 46–48^. For clarity, we in the following adopt the Caputo calculus, whose definition is given in Methods. Note that the traditional Maxwell model is restored by replacing *α* with 1 in equation (1). Moreover, under the condition of constant axial stress, the time dependent creep compliance *J*(*t*) of the fractional Maxwell model is expressed as follows:

2 $$J(t)=\frac{1}{E}+\frac{1}{{\eta }^{\alpha }}\frac{{t}^{\alpha }}{{\rm{\Gamma }}(1+\alpha )}$$

where Γ is the Gamma function, whose definition can be found in Methods. Again, the creep compliance of the Maxwell model is obtained by replacing α with 1 in equation (2). On the other hand, as α approaches 0, both *η* and *η* ^*α*^ will tend to infinity, so that the time-dependent deformation becomes vanishingly small, and the Hooke’s law for pure elasticity *ε* = *σ*/*E* will be restored.

For each of the four rheological models considered in this work, we estimate the parameters in the corresponding *J*(*t*), respectively. In Table 1, all the *J*(*t*)’s, as well as the estimated parameters and the goodness of fit *R* ^2^ are listed. Although *R* ^2^ is as high as 0.99 in each case, we can see visually in Fig. 2 that the fractional Maxwell model captures the full trend of the experimental data in a more accurate manner than all the other three models.

In Fig. 2, the experimental results of the H20-6 sample are fit to the four rheological models aforementioned. Our first observation is that the traditional Maxwell model is not proper for the description of the creep behaviour of tight sandstone. The Maxwell model predicts a constant creep rate, hence a linear relation between strain ε and time *t* is expected. Nonetheless, ε is apparently nonlinear in the whole range of experimental time, indicating the failure of the Maxwell model in this case. That is to say, the underlying creep dynamics of tight sandstone is not a process with a simple relaxation mode. One can consider more complex models and expect more accurate predictions, however, at the expense of more parameters to be estimated. The three-parameter generalized Kelvin and Burgers models generalize the Maxwell model by introducing one and two more parameters, respectively, and it is not surprising that their performances are enhanced as the number of model parameters is increased. In particular, the Burgers model takes into account two relaxation modes and thus makes more realistic predictions than the Maxwell model. But even for the Burgers model, there is still an obvious discrepancy between the theoretical prediction and the experimental data at early times, see Fig. 2. In our case, it means the creep dynamics of tight sandstone can be much more complicated than naively considered, possibly involving a large amount of relaxation modes. If we approximately take the number of relaxation modes to infinity, then we are not arriving at a model with an infinite number of parameters, rather, we are confronted with a model that minimally possesses only three parameters, i.e., the fractional Maxwell model. It is visually evident that the prediction based on such a model almost perfectly agrees with the experimental data in the full range of time, outperforming all other three models. We believe the success of the fractional Maxwell model provides strong evidence that the creep dynamics can be history dependent for tight sandstone.

**Figure 3:**
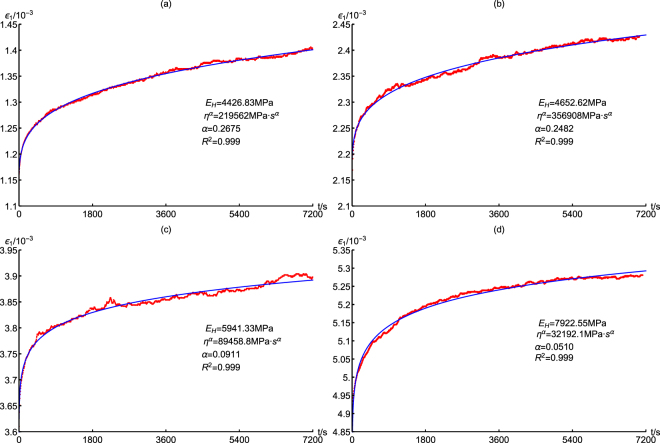
Fitting experimental data of all samples to the fractional Maxwell model. (**a**) Sample H20-6 under 5 MPa axial stress. (**b**) Sample C3-2 under 10 MPa. (**c**) Sample G5-6 under 20 MPa. (**d**) Sample W1-1 under 30 MPa. For each rock sample, the predicted value (blue line) of the axial strain ε is plotted, and they are all in good agreement with experimental data (red dot).

We also emphasize that the superiority of the fractional Maxwell model to other three models can be observed not only for the H20-6 rock sample case; it persists in all our four rock sample cases that display viscoelastic deformation. In Fig. 3, the predicted ε by the fractional Maxwell model is plotted along with the experimental data for each sample. In each case, we observe satisfactory match between theory and experiment.

### Prediction of long-term strain rate by the fractional Maxwell model

We have shown above that the fractional Maxwell model can be used to essentially reproduce our experimental data. However, the laboratory creep experiment is inevitably subject to relatively short experimental time. Therefore, before we can propose this model as a reasonably reliable approach to modelling tight sandstone deformation under *in situ* stress conditions, we should check its long-term consistency with both existing experimental data and theoretical estimations.

**Figure 4:**
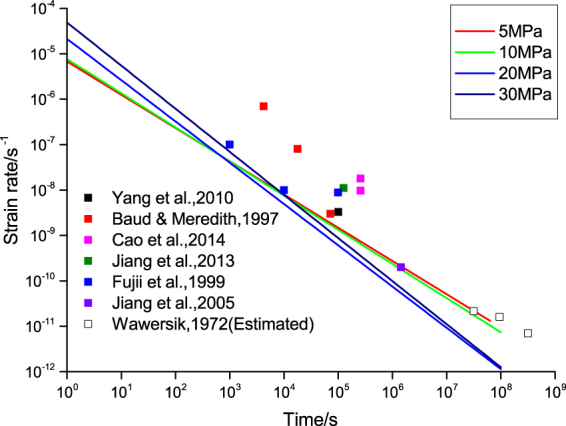
Comparison of the strain rates. The strain rate of tight sandstone^49–54^ obtained from the fractional Maxwell model is plotted against time for each sample. It is represented by a straight line on a log-log scale as a result of its power-law dependence on time. Also plotted are various experimental data (filled squares) and estimations (squares) for general sandstone (not necessarily tight sandstone). The factional Maxwell prediction is consistent with these results up to several years; the observation that it seems to reach some lower bound of sandstone strain rate is also in accord with the fact that tight sandstone is typically “harder” than many other kinds of ordinary sandstone.

To this end, we systematically compare the strain rate of tight sandstone obtained from the fractional Maxwell model and those of other kinds of sandstone obtained from both experiments and theoretical considerations. Based on equation (2), the axial creep strain rate $\dot{\varepsilon }(t)$ under constant load is

3 $$\dot{\varepsilon }(t)=\dot{J}(t)\sigma =\frac{\alpha \sigma }{{\eta }^{\alpha }{\rm{\Gamma }}(1+\alpha )}{t}^{\alpha -1}$$

which decreases in a power-law manner (note that 0 ≤ *α* ≤ 1)). Also note that the traditional Maxwell model is recovered when *α* = 1, and in this case $\dot{\varepsilon }(t)$ is indeed a constant. We in Fig. 4 plot the strain rate predictions from both the fractional and the traditional Maxwell models. Also plotted are various experimental results for sandstones (not necessarily tight sandstone) that span a time interval from several hours to more than a month. We are not able to find experimental results up to several years, however, there have been estimations about sandstone’s long-term strain rate. The prediction from the fractional Maxwell model agrees well with all these experimental results and estimations. It is also worth noting that since tight sandstone is typically “harder” than other kinds of ordinary sandstone, its predicted strain rate seems to reach the lower bound of the strain rate of sandstone as a whole.

With such long-term qualitative consistency with previous independent findings, as well as with its capability of quantitative interpretation of our laboratory data, we believe the fractional Maxwell model is thus reasonably qualified to serve as a basis for the analysis of tight sandstone creep behaviour. In hydraulic fracturing, it is key to maintain the high fracture conductivity. However, if rock deforms in a viscoelastic way, then the fracture will close under the constant stress condition and the conductivity will decrease. Our work can be used to simulate this process and help to design the re-stimulation plan.

### Stress relaxation process

Having justified its validation, here we use the fractional Maxwell model to analyze the stress relaxation of tight sandstone. A key quantity that characterizes the stress relaxation process is the relaxation modulus *G*(*t*). We also omit the mathematical details and only demonstrate our main theoretical results here. *G*(*t*) can be obtained as

4 $$G(t)=E\cdot {{\rm{E}}}_{\alpha ,1}(-\frac{E}{{\eta }^{\alpha }}{t}^{\alpha })$$

where E_*α*_,_1_ is the generalized Mittag-Leffler function^55–57^, see Methods for its definition.

As a comparison, we also calculate the relaxation modulus of the Maxwell model by replacing α in equation (4) with 1. Note that E_1,1_(*x*) = exp(*x*), we have

5 $$G(t)=E\cdot \exp (-Et/\eta )$$

**Figure 5:**
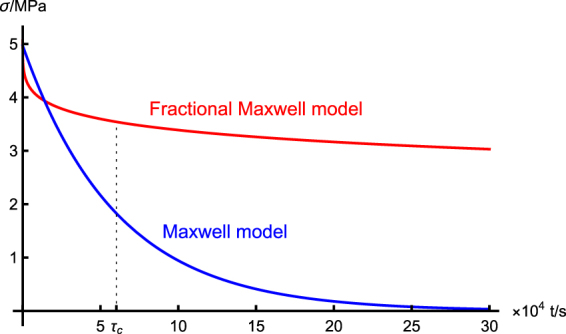
Stress relaxation process of the H20-6 sample as predicted by the fractional and traditional Maxwell models, respectively. For the latter, there is a characteristic time scale *τ*_*c*_, and the stress rapidly decreases towards a vanishing value when *t* ≥ *τ*_*c*_. On the contrary, the former undergoes a much slower rate of decrease at later times.

Clearly, the relaxation modulus obtained from the fractional Maxwell is qualitatively different from that obtained from the Maxwell model. Under the condition of constant strain, the latter indicates an exponential decrease of stress, while the former corresponds to a much slower rate of decrease. This means for the latter, there exists a definite time scale *τ*_*c*_ = *η*/*E*, and when *t* ≥ *τ*_*c*_, the stress rapidly becomes negligibly small. Contrary to it, in the former case, there is no such characteristic time scale, and the stress decrease at a much slower pace, hence it will keep at a relatively high level for a long time. As an example, in Fig. 5, we plot the stress relaxation process for the H20-6 sample, based on the fractional and traditional Maxwell model, respectively. At the early stage of the relaxation process, the fractional Maxwell model predicts a larger stress relaxation than the traditional Maxwell model; while in the following time, the Maxwell model outpaces its fractional counterpart. We ascribe this observation to the multi-mode nature of the fractional Maxwell model. At later times, the contribution from slow modes dominates the relaxation process. The single-mode Maxwell model, however, fails to predict this slowing-down relaxation process. For other samples, we find similar observations and we do not show them here.

As hydraulic fracturing is operated, high pressure fluids with proppants are injected into the tight sandstone. When the fracture network is created, the fluids are recovered and a layer of proppants will be left in the subsurface to keep the fracture open. Consider for a segment of a fracture with proppant support, the normal strains of the fracture’s two surfaces are essentially fixed, then equation (5) is supposed to help calculate the instantaneous stress level of this part of the fracture. (Of course, we have to make some assumptions to simplify the realistic *in situ* situations which are formidably complicate. For example, we will assume the axial stress *σ* in our work can be interpreted as the differential stress when the circumferential stress is present). As the stress is relaxed, this part of the fracture will possibly deform and consequently push the adjacent portion of the rock without proppant support to expand, i.e., the un-supported segment of the fracture is likely to close as a result of the stress relaxation process of the rock with proppant support. Fracture closure in this way is complementary to that purely due to stress-induced strain increase as studied above. Our work thus provides a reasonably reliable approach to modelling the long-time behaviour of the fracture-closure process that is pivotal to tight gas recovery. Relaxation models can also be utilized to estimate the stress magnitude as a function of depth in sedimentary formations, as proposed by Sone and Zoack^58^. They built a reasonable profile of the principal stress magnitudes from the geophysical well logs by their stress relaxation model^59^. Our stress relaxation model can also provide the variations of vertical stress from well logs, which would be of great help in identifying the intervals that could (or could not) serve as barriers during hydraulic fracturing. Nonetheless, these are beyond the scope of this work, and we will leave them for future study.

## Discussion

In our work, we have performed creep experiments for four samples of tight sandstone from the Changqing oilfield in China. The unexpected creep behaviour is observed in each case. One question one might ask is whether the results of our experiments are typical. Although it is unlikely for us to perform such an experiment to each sample that we collect and then assess the typicality of the tight sandstone’s viscoelasticity property, we can nevertheless gain some useful information indirectly and make plausible estimations accordingly.

**Figure 6:**
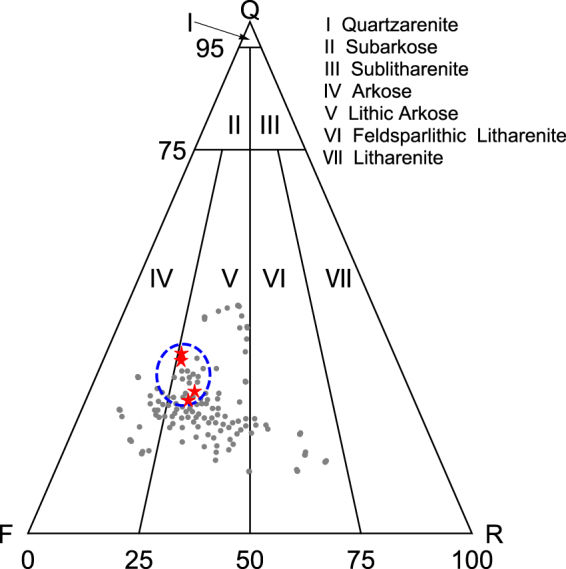
A widely used triangle classification map of sandstone in the Changqing oilfield. The gray dots represent the data of 122 samples in a previous study^60^, and we highlight our 4 sample points with red stars. There are almost 22 gray dots near the stars. Partly based on this fact, we estimate the viscoelasticity property is likely to be possessed by approximately 18% samples.

The mechanical properties of a certain sample mainly rely on two factors: its mineral contents and the geometry of the pore space. Prior to the creep experiments, the mineral contents of four samples are quantified by the XRD (X-ray diffraction) test, and the results are shown in Table 2. Are our samples typical in contents? We notice there has been a systematical content analysis conducted for 122 tight sandstone samples in the same oilfield^60^. Then, a widely used triangle classification map of sandstone in this region is adopted to demonstrate the results, and we highlight our four sample points on the map. See Fig. 6. It turns out that the samples we use to perform creep experiments are lithic arkose, which is the most common sandstone type in this area. Hence, from the mineral content perspective, our samples are definitely not outliers and do represent some portion of the tight sandstone regionally.

**Table 2:** Mineral contents of four tight sandstone samples.

Sample	Clastic Contents				Filling Contents/%
	Quartz/%	Feldspar/%	Lithite/%	Others/%	
H20-6	30.1	43.0	10.2	5.3	11.4
C3-2	22.6	43.9	12.6	7.3	13.6
G5-6	23.0	39.9	10.2	9.2	17.7
W1-1	31.2	42.1	9.3	5.6	11.8

We further know that the porosities of the sandstone samples in this area are narrowly distributed between 5.0~11.0% with an average of 7.9%. To the lowest order of approximation, i.e., neglecting more complex structural features such as the spatial variation and/or correlation of porosity, we can roughly say the sandstone in this region is very weakly heterogeneous. That is to say, our samples are also reasonably typical in structure.

We are now at a stage to evaluate how typical our experimental results are. There are roughly 22 sample points in the vicinity of our sample points on the classification map, then based on the reasoning above, we estimate that roughly 18% tight sandstone samples are expected to behave (more or less) in a viscoelastic manner. Thus, the viscoelasticity should be taken into account when modelling the tight sandstone’s mechanical properties in the Changqing oilfield.

Moreover, the results presented in this work also suggest that a further scrutiny is necessary for materials whose mechanical properties are conventionally taken for granted as simply elastic. Just like that we do not expect the tight sandstone’s viscoelasticity until we find it experimentally, it is also possible that some other materials, not necessarily rock, will behave in an unexpected way that can only be uncovered by direct experimentation rather than intuitive perception.

## Methods

### Experimental Procedure

**Figure 7:**
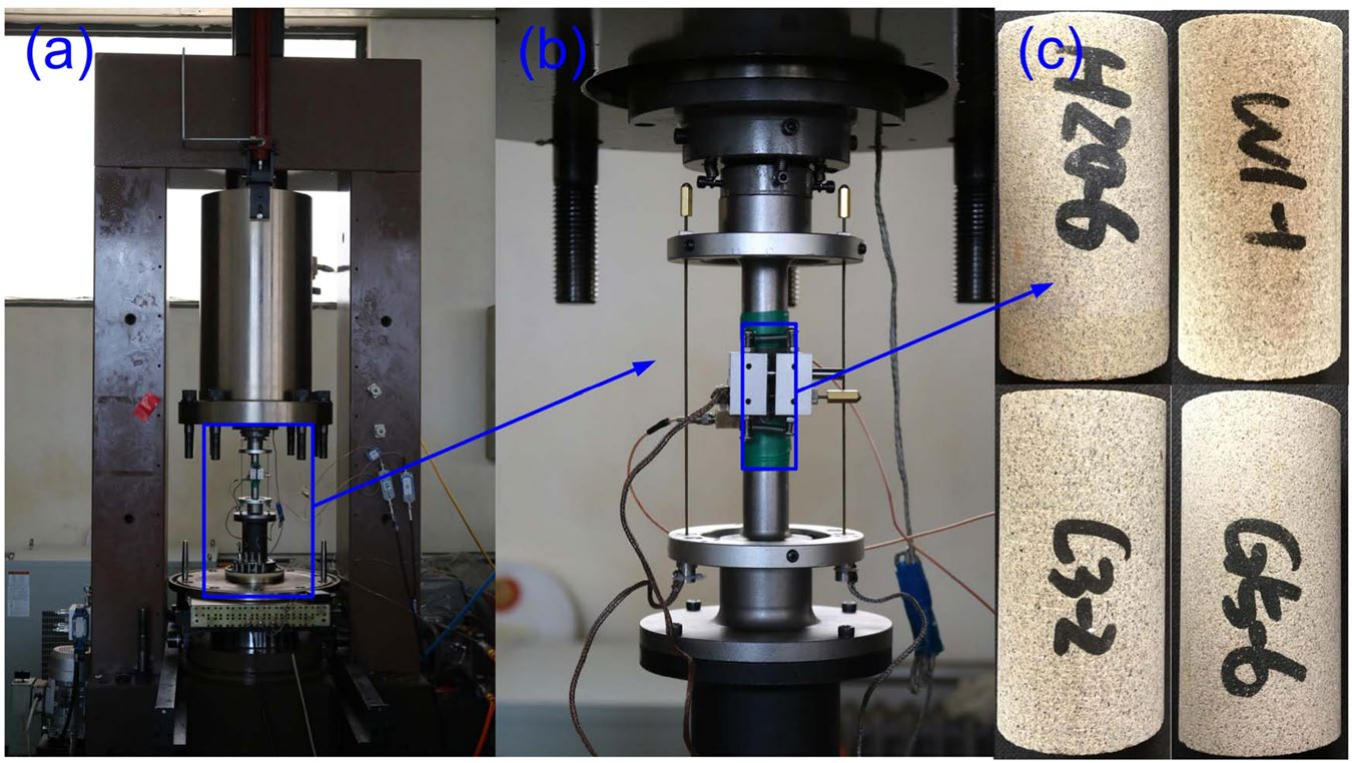
Servo-controlled rock testing apparatus. (**a**) The servo-controlled rock testing machine that is used to conduct our experiments. **(b)** A pair of LVTD displacement transducers installed to record the axial deformation and a chain type strain transducer used to measure lateral deformation. **(c)** Tight sandstone samples.

Four cylindrical samples for uniaxial creep experiments are prepared first, each with diameter of 25 mm and length of 50 mm. Each of these samples is drilled from a sandstone core with diameter 100 mm, which is obtained from a tight sandstone reservoir in the Changqing oil field, China. The samples are homogeneous, isotropic, and free of bedding and cracks in appearance. The uniaxial creep experiments are conducted with a servo-controlled apparatus in the rock mechanics laboratory at China University of Petroleum, Beijing. A pair of LVTD displacement transducers is installed to record the axial deformation, and a chain type strain transducer is used to measure the lateral deformation. See Fig. 7 for the pictures of sandstone samples and the experimental apparatus.

Four experiments are conducted, and in each of them the creep behaviour of one sample is measured under a given axial stress. Four axial stresses are set to be 5 MPa, 10 MPa, 20 MPa, and 30 MPa, respectively. There is no circumferential stress. In each experiment, the axial stress is rapidly increased from 0 to the predetermined value, and then kept as a constant for 7200s. All the experiments are conducted under room-dry, room-temperature conditions. The loading stage, in which the axial stress is increased, always lasts for less than 50 seconds. Hence it is negligibly short compared with the total experiment time, and the sample’s viscoelastic deformation in this stage is small and neglected when fitting data to the rheological models considered in this work.

### Constitutive equation of the fractional Maxwell model

The constitutive equation of ideal elastic materials is described by the Hooke’s law, which states that strain and stress are linearly correlated. While for the Newtonian fluid, stress is a linear function of the rate of strain, rather than strain itself, and this is the so-called Newton’s law: ${\rm{\sigma }} \sim {{\rm{d}}}^{1}{\varepsilon }_{{\rm{v}}}/{\rm{d}}{t}^{1}$, where the superscript 1 is used to highlight the fact that the order of time derivative here is 1, and the subscript ${\rm{v}}$ denotes that strain is time dependent. Similarly, we can also formally rewrite the Hooke’s law as ${\rm{\sigma }} \sim {{\rm{d}}}^{0}{\varepsilon }_{{\rm{v}}}/{\rm{d}}{t}^{0}$, where the superscript 0 denotes the order of time derivative is 0 (indeed there is no differentiation and strain is time independent). For materials whose time-dependent deformation behaviour is in between these two cases, it is natural to formally propose the corresponding constitutive relation as ${\rm{\sigma }} \sim {{\rm{d}}}^{\alpha }{\varepsilon }_{{\rm{v}}}/{\rm{d}}{t}^{\alpha }(0 < \alpha  < 1)$. The superscript α denotes the order of the fractional derivative, and the operator ${d}^{\alpha }/d{t}^{\alpha }$ is understood in the sense of Caputo^61^, which is generally defined as ${d}^{\alpha }f(t)/d{t}^{\alpha }\equiv$ ${}_{0}^{C}{D}_{t}^{\alpha }f(t)=\frac{1}{{\rm{\Gamma }}(n-\alpha )}{\int }_{0}^{t}\frac{{f}^{(n)}(\tau )d\tau }{{(t-\tau )}^{\alpha -n+1}}$, where *n* is an integer and $n-1\le \alpha  < n$. The symbol Г is the Gamma function^62^, defined as ${\rm{\Gamma }}(z)={\int }_{0}^{\infty }{e}^{-t}{t}^{z-1}{\rm{d}}t$. Mathematically, the fractional order time derivative can be understood via the Laplace transform: the operator ${{\rm{d}}}^{\alpha }/{\rm{d}}{t}^{\alpha }$ is essentially transformed to ${s}^{\alpha }$, where s is the complex frequency in the Laplace domain. Then the constitutive equation in the Laplace domain is $\sigma (s) \sim {s}^{\alpha }{\varepsilon }_{{\rm{v}}}(s)$. Physically, the fractional time derivative plays a central role in a good approximation of some process-specific memory kernel function *M*(*t*)^63^. To see this, let us consider the most general situation that stress at some time is related to the whole history of the process. We can write $\sigma (t)={\int }_{0}^{t}M(t-\tau ){\varepsilon }_{{\rm{v}}}(\tau ){\rm{d}}\tau$, and then by Laplace transform, we have $\sigma (s)=M(s){\varepsilon }_{{\rm{v}}}(s)$. However, the precise form of $M(t)$ usually is not known because it is not likely to gain the information of the process in question in a quantitative way. Besides that, in many times it is also unnecessary to know the details of the process, especially when we are only interested in the system’s long time behaviour of deformation response to external driving, which is reflected in the scaling behaviour of $M(t)$ in the long time limit, or equivalently, in that of $M(s)$ in the small s limit. Therefore, if it turns out that $M(s) \sim {s}^{\alpha }$ for small s, then the general stress-strain relation can be well approximated by the fractional constitutive equation. Not surprisingly, due to its close relation with the memory kernel $M(t)$, the fractional derivative can be utilized as a powerful tool for describing time-dependent deformations. Similar reasoning also leads to the fractional derivative’s application in other situations. For example, fractional advection-diffusion equations have become one popular approach to modelling non-Fickian transport in biological and geological systems^64–66^.

Now, let us derive the constitutive equation of the fractional Maxwell model, which is graphically represented by a Hooke spring and a Scott Blair dashpot that are connected in serial; see the inset of Fig. 1. The Scott Blair dashpot is an element which describes the rheological behaviour of materials in between elastic solid and Newtonian fluid. Unlike the classical integer order dashpot that is characterized only by one damping parameter ${\rm{\eta }}$, the Scott Blair dashpot is characterized by two parameters ${\rm{\eta }}$ and ${\rm{\alpha }}$. For given stress ${\rm{\sigma }}$, the corresponding strain ${\rm{\varepsilon }}$ can be divided into two parts:$\,\varepsilon ={\varepsilon }_{{\rm{e}}}+{\varepsilon }_{{\rm{v}}}$, where ${\varepsilon }_{{\rm{e}}}$ denotes the elastic strain which is time independent, and ${\varepsilon }_{{\rm{v}}}$ as above denotes the time-dependent deformation. We know ${\varepsilon }_{{\rm{e}}}=\sigma /E$ as a result of the Hooke’s law, where $E$ is Young’s modulus. For the time-dependent ${\varepsilon }_{{\rm{v}}}$, a fractional constitutive equation $\sigma ={\eta }^{\alpha }{{\rm{d}}}^{\alpha }{\varepsilon }_{{\rm{v}}}(t)/{\rm{d}}{t}^{\alpha }\,$can be established. Combing these results together, and after some algebra, we arrive at the fractional Maxwell model’s constitutive equation (1).

Based on it, two quantities that are of fundamental importance in characterizing the fractional Maxwell model’s response dynamics can be derived. The first is the time-dependent creep compliance $J(t)$ under the condition of constant axial stress, which is defined via $\varepsilon (t)={\int }_{0}^{t}J(t-\tau ){\rm{d}}\sigma (\tau )$. In our experimental setting, the loading period is very short, then we have $\,\sigma (t)\approx \sigma {\rm{\Theta }}(t)$, where ${\rm{\sigma }}$ denotes one of the prescribed constant axial stress and ${\rm{\Theta }}(t)$ is the Heaviside step function. The time derivative of ${\rm{\Theta }}(t)$ is the Dirac delta function $\delta (t)$. Therefore, we find $J(t)=\varepsilon (t)/\sigma$. Then by Laplace transform of equation (1), we know in the Laplace domain $\,J(s)=1/(Es)+$ $1/({\eta }^{\alpha }{s}^{1+\alpha })$. Note that the Laplace transform of ${t}^{q}\,(q > -1)$ is $\Gamma (1+q)/{s}^{1+q}$, then the expression (2) of $J(t)$ is obtained directly by inverse Laplace transform of $J(s)$. In our work,$\,J(t)$ is used to help estimate the parameters in the fractional Maxwell model; see Table 1.

The other quantity of interest is the time-dependent relaxation modulus $G(t)$ under the condition of constant strain, which is defined via $\sigma (t)={\int }_{0}^{t}G(t-\tau ){\rm{d}}\varepsilon (\tau )$. Similarly, in this case $\varepsilon (t)\approx \varepsilon {\rm{\Theta }}(t)$ with $\,\varepsilon$ being a constant, and we have in the Laplace domain that $\,G(s)={s}^{\alpha -1}/({s}^{\alpha }/E+1/{\eta }^{\alpha })$. To obtain $G(t)$, however, let us rewrite $G(s)$ as $G(s)=A(s)E/s$, where $\,A(s)={(1+E/{\eta }^{\alpha }{s}^{\alpha })}^{-1}={\sum }_{n=0}^{\infty }{(-E/{\eta }^{\alpha }{s}^{\alpha })}^{n}$. Then $\,G(s)$ can be expressed in terms of an infinite series as $\,G(s)=E{\sum }_{n=0}^{\infty }{(-E/{\eta }^{\alpha })}^{n}{s}^{-\alpha n-1}$, whose inverse Laplace transform is $\,G(t)=$ $E{\sum }_{n=0}^{\infty }{(-E{t}^{\alpha }/{\eta }^{\alpha })}^{n}/{\rm{\Gamma }}(\alpha n+1)$. Taking advantage of the Mittag-Leffler function ${{\rm{E}}}_{\alpha ,\beta }(z)$, which is defined as ${{\rm{E}}}_{\alpha ,\beta }(z)\equiv {\sum }_{n=0}^{\infty }{z}^{n}/{\rm{\Gamma }}(\alpha n+\beta )$, we finally reach $\,G(t)=E\cdot {{\rm{E}}}_{\alpha ,1}(-E{t}^{\alpha }/{\eta }^{\alpha })$, and this is equation (4). We in this work use *G*(*t*) to predict the stress relaxation process of our tight sandstone sample; see Fig. 4.

### Data availability statement

All raw data used in this work are available upon request to Guangqing Zhang (email: zhangguangqing@cup.edu.cn).
